# Production, Quality Control and Pharmacokinetic Studies of ^166^Ho-EDTMP for Therapeutic Applications

**DOI:** 10.3797/scipharm.1004-21

**Published:** 2010-06-09

**Authors:** Ali Bahrami-Samani, Reza Bagheri, Amir R. Jalilian, Simindokht Shirvani-Arani, Mohammad Ghannadi-Maragheh, Mojtaba Shamsaee

**Affiliations:** 1 Radiopharmaceutical Research and Development Lab (RRDL), Nuclear Science and Technology Research Institute (NSTRI), Tehran, Postal code: 14155-1339, Iran; 2 Faculty of Nuclear Engineering and Physics, Amir Kabir University, Tehran, Iran

**Keywords:** Holmium-166, EDTMP, Radiopharmaceutical therapy, Biokinetic

## Abstract

^166^Ho-EDTMP is a major therapeutic agent which is widely used in bone palliation therapy. In this study, a ^166^Ho-EDTMP complex was prepared successfully using an in-house synthesized EDTMP ligand and ^166^HoCl_3_. Ho-166 chloride was obtained by thermal neutron irradiation (1 × 10^13^ ncm^−2^s^−1^) of natural Ho(NO_3_)_3_ samples (specific activity = 3–5 GBq/mg), dissolved in acidic media. The radiochemical purity of ^166^Ho-EDTMP was checked by ITLC (>99%) and stability studies in presence of human serum and final preparation were performed. The biodistribution of ^166^Ho-EDTMP and ^166^HoCl_3_ in wild-type rats was checked by scarification. SPECT imaging of ^166^Ho-EDTMP was also performed in wild-type rats. A comparative accumulation study for ^166^Ho-EDTMP and ^166^HoCl_3_ was performed for vital organs up to 48h. Significant bone accumulation (>70%) of the tracer in 48h was observed.

## Introduction

Bone metastases are common in the progression of various tumors such as prostate, breast, and lung carcinoma and they often entail an occurrence of progressive pain [1] and occur in many patients with solid malignant tumors [2]. Approximately 50% of patients with breast carcinoma and 80% of patients with prostate carcinoma develop metastatic bone diseases and nearly half of them experience bone pain [3]. In these patients who have progressive disease despite treatment, a systemic bone-avid radiopharmaceutical for treatment of widespread bone metastases has potential benefits [4].

Multidentate polyaminopolyphosphonic acid ligands are known to form stable chelates with many metals including lanthanides. Among them, ethylenediaminetetramethylene phosphonic acid (EDTMP) can be envisaged as an ideal carrier moiety, for the development of beta emitter-based radiopharmaceuticals, for bone palliation.

Many beta-emitters such as Sm-153, Lu-177 and Ho-166 can be produced in reasonable amounts using (n, gamma) reactions. Holmium-166 (E_β_^−^ max = 1.84 MeV, T_1/2_ = 26.8 hr) is an interesting radionuclides for targeted therapy modalities. Although it is not available in high specific activities, but the uni-elemental abundance makes it an accessible and inexpensive radionuclide and obtained specific activity is enough for radiolabeling of small molecules at radiopharmaceutical grades.

Various therapeutic bone-seeking agents have been reported and used in human studies including ^153^Sm-EDTMP (Lexidronam) [5], ^177^Lu-EDTMP [6] and ^166^Ho-DOTMP [7], among those, ^153^Sm-EDTMP is the most widely used compound in the world. We have recently reported the production and human application of this compound in the country [8].

^166^Ho-EDTMP has been reported as a bone seeking therapeutic radiopharmaceutical for therapeutic applications, however its high beta energy leading to bone marrow toxicity makes it an interesting specific marrow ablation agent before marrow transplantation [9].

Although the canine biodistribution and marrow toxicity of this radiopharmaceutical has been reported [9], no biodistribution data has been reported on rat animals.

The rat animal biodistribution data is important since it is possible to estimate the absorbed radiation dose to human organs following intravenous administration of radio-pharmaceutical by using the distribution data for normal rats [10]. These data are mandatory before any human trial studies.

In this research, ^166^Ho-EDTMP complex was prepared from in-house made starting ligand and biokinetic studies of the compound was investigated among vital rat organs by scarification studies and imaging for future human studies in the country.

## Results and discussion

### Production and quality control of ^166^Ho

**Fig. 2.**
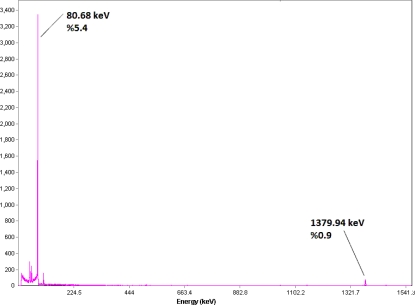
Gamma spectrum for ^166^HoCl_3_ solution used in the radiolabeling

The radionuclide was prepared in a research reactor according to regular methods with a range of specific activity 3–5 GBq/mg for radiolabeling use, after counting the samples on an HPGe detector for 5 min and two major photons (5.4% of 80.68 keV and 0.9% of 1379.94 keV) were observed. The radioisotope was dissolved in acidic media as a starting sample and was further diluted and evaporated for obtaining the desired pH and volume followed by sterile filtering (Figure 2).

Radiochemical impurities in the ^166^Ho sample used in the radiolabeling step were checked by two solvent systems; A, a mixture of 10 mM DTPA solution as mobile phase on Whatman No.1 paper (pH.3), the free holmium cation in ^166^Ho^3+^ form, was chelated with the polydentate compound leading to the migration of the cation in ^166^Ho-DTPA form to higher R_f_ (R_f_.0.9), any other ionic species (such as ^166^HoCl_4_^−^, etc.) would lead to the observation of new radiopeaks, especially at the origin (R_f_.0.0–0.1). B, a mixture of 10% ammonium acetate:methanol (1:1) was used as another solvent system on the Whatman No,.1 paper, ^166^Ho^3+^ remains at the origin using this system while other ionic species would migrate to higher R_f_s.

### Radiolabeling

To investigate the effect of EDTMP concentration on labeling yield various amounts of the ligand were added to fixed amount of activity. Labeling yield increased with increasing ratio amount (from 1:5 to 1:15) and reached above 99% in 60 minutes.

**Fig. 3.**
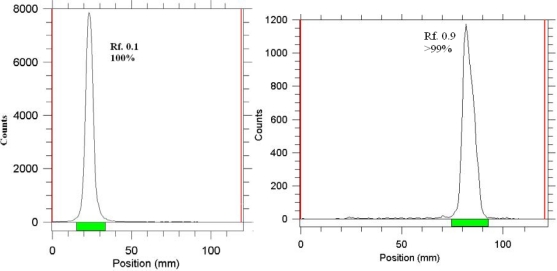
ITLC chromatograms of ^166^HoCl_3_ (left) and ^166^Ho-EDTMP solution (right) using Whatman 1 MM eluted with NH_4_OH: MeOH: H_2_O (0.2:2:4).

Figure 3 demonstrates the radiochromatograms of free Ho-166 and radiolabeled product at optimized conditions in NH_4_OH:MeOH:H_2_O (0.2:2:4) solvent system. The stability of prepared ^166^Ho-EDTMP complex was checked up to 48 hours after preparation. The complex was stable in final sample and its radiochemical purity was above 99% even 48 hours after preparation using Whatman 1 MM eluted with NH_4_OH: MeOH: H_2_O (0.2:2:4). Stability test was developed for the complex in presence of human serum at 37°C using ITLC as mentioned above and also no change of radiochemical yield was observed for 24h.

### Biodistribution Studies

The animals were sacrificed by CO_2_ asphyxiation at selected times after injection (2, 3, 4, 24 and 48h).

**Fig. 4.**
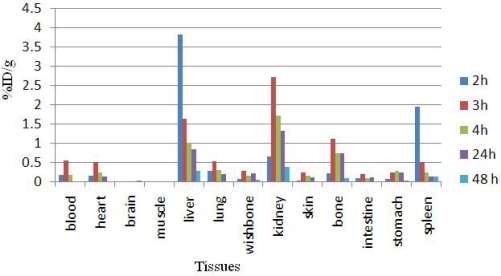
Percentage of injected dose per gram (ID/g %) of ^166^HoCl_3_ in rat tissues at 2, 3, 4, 24 and 48 h post injection

**Fig. 5.**
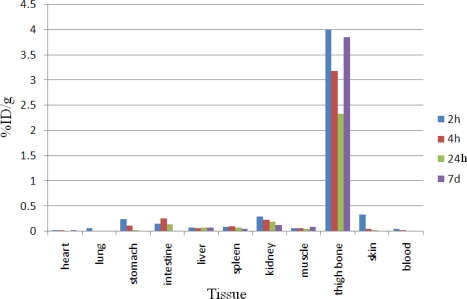
Percentage of injected dose per gram (ID/g %) of ^166^Ho-EDTMP in wild-type rat tissues at 2, 4, 24h and 7d post injection

Dissection began by drawing blood from the aorta followed by removing heart, spleen, muscle, brain, bone, kidneys, liver, intestine, stomach, lungs and skin samples. The tissue uptakes were calculated as the percent of area under the curve of the related photo peak per gram of tissue (% ID/g) (Fig. 4). For ^166^Ho^3+^ cation, the radioactivity was mainly located in the liver, kidney and bone. The free cation is soluble in water and it can be excreted *via* the urinary tract. Since the metallic ^166^Ho is transferred in plasma into a protein-bond form, the major final accumulation was shown to be in the liver. The distribution of injected dose in rat organs up to 7d after injection of ^166^Ho-EDTMP (200 μCi/150ul) solution was determined. Based on these results, it was concluded that the major portion of injected activity of ^166^Ho-EDTMP was extracted from blood circulation into bones (Figure 5).

**Fig. 7.**
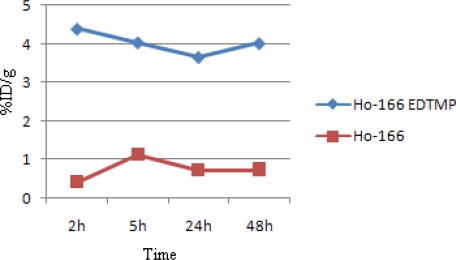
Comparative bone activity for ^166^Ho-EDTMP and ^166^HoCl_3_ in wild-type rats

For better comparison of the ^166^Ho-EDTMP and ^166^HoCl_3_ species behavior, Fig. 7 demonstrates the blood accumulation from 2 to 48h. Both compounds are washed out from the circulation after 48 h, although the blood wash-out mechanisms are different.

Fig. 7 demonstrates the bone accumulation from 2 to 48h. ^166^Ho-EDTMP is rapidly taken up in bones in 2h after administration and retains almost constantly up to 24 h. Instead, ^166^Ho cation uptake slowly increases but never exceeds %1.

**Fig. 8.**
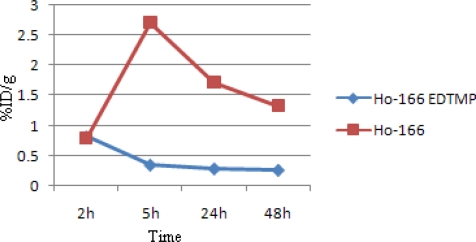
Comparative kidney activity for ^166^Ho-EDTMP and ^166^HoCl_3_ in wild-type rats

Fig. 8 demonstrates kidney activity from 2 to 48h. As mentioned earlier, ^166^Ho-EDTMP is rapidly taken up in bones and the trapping continued in a way that almost no blood circulation activity as well as kidney excretion can be observed. Instead, as a water soluble cation most of free Ho-166 activity is washed out through kidney in 48h.

**Fig. 9.**
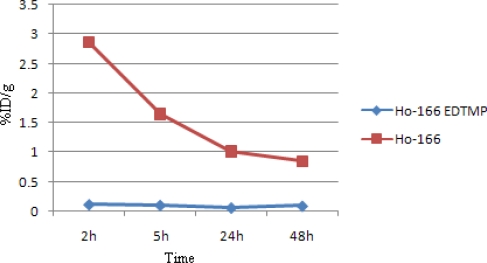
Comparative liver activity for ^166^Ho-EDTMP and ^166^HoCl_3_ in wild-type rats

A major difference in liver uptake is observed for two species. Fig. 9 demonstrates liver accumulation from 2 to 48h. ^166^Ho-EDTMP has almost no liver accumulation, which is a major advantage as a therapeutic radiopharmaceutical due to the possibility of increasing the maximum administered dose compared to other bone seeking therapeutic radiopharmaceuticals such as ^177^Lu-EDTMP and ^153^Sm-EDTMP. While Ho^3+^ cation, being transferred by serum metalloproteins, accumulates in liver and is excreted through hepatobilliary excretion route, leading to the reduction in liver accumulation.

**Fig. 10.**
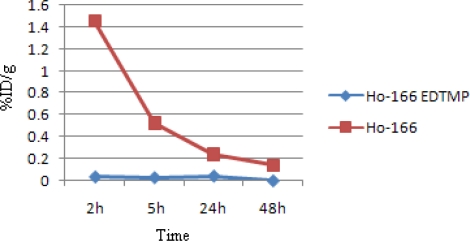
Comparative spleen activity for ^166^Ho-EDTMP and ^166^HoCl_3_ in wild-type rats

Also, a major difference in spleen uptake is observed for the two species as shown in Fig.10. ^166^Ho-EDTMP almost is not accumulated in spleen which can be again a major advantage as a therapeutic radiopharmaceutical due to the possibility of increasing the maximum administered dose, while Ho-166 cation is present in spleen 2h post injection while slowly is washed out in 48h.

### Imaging study

**Fig. 11.**
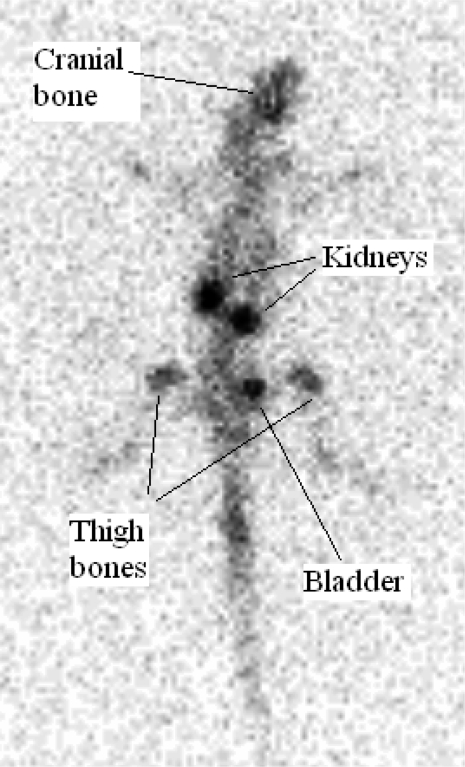
Planar scintigraphic images of ^166^Ho-EDTMP 4 h post injection in wild-type rat

As shown in figure 11, the complex is majorly washed out from the circulation in first few hours through kidneys while is also trapped in bones especially in vertebra, cranial and thigh bones and insignificant activity is accumulated in other tissues.

## Experimental

Production of ^166^Ho was performed at the Tehran Research Reactor (TRR) using ^165^Ho (n, gamma)^166^Ho nuclear reaction. Natural holmium nitrate with purity of >99.99% was obtained from Merck Co. Whatman No. 1 was obtained from Whatman (Maidstone, UK). Radio-chromatography was performed by using a Bioscan AR-2000 radio TLC scanner instrument (Bioscan, Paris, France). A high purity germanium (HPGe) detector coupled with a Canberra™ (model GC1020-7500SL) multichannel analyzer and a dose calibrator ISOMED 1010 (Dresden, Germany) were used for counting distributed activity in rat organs. All other chemical reagents were purchased from Merck (Darmstadt, Germany). Calculations were based on the 80.6 keV peak for ^166^Ho. Animal studies were performed in accordance with the United Kingdom Biological Council's Guidelines on the Use of Living Animals in Scientific Investigations, 2nd edn. Male healthy rats were purchased from Pasteur Institute, Tehran, Iran.

### Production and quality control of ^166^HoCl_3_ solution

Holmium-166 was produced by neutron irradiation of 1000 μg of natural ^165^Ho(NO_3_)_3_ (^165^Ho, 99.99% from Merck Co.) according to reported procedures [11] at the Tehran Research Reactor at a thermal neutron flux of 4×10^13^ ncm^−2^s^−1^. Specific activity of the produced ^166^Ho was 5 GBq/mg after 20h of irradiation. The irradiated target was dissolved in 200 μl of 1.0 M HCl, to prepare ^166^HoCl_3_ and diluted to the appropriate volume with ultra pure water, to produce a stock solution. The mixture was filtered through a 0.22 μm filter (Millipore, Millex GV) and sent for use in the radiolabeling step. The radionuclidic purity of the solution was tested for the presence of other radionuclides using beta spectroscopy as well as HPGe spectroscopy for the detection of various interfering beta and gamma emitting radionuclides. The radiochemical purity of the ^166^HoCl_3_ was checked using 2 solvent systems for ITLC (A: 10mM DTPA pH.4 and B: ammonium acetate 10%:methanol (1:1)).

### Synthesis of [Ethane-1,2-diylbis(nitrilodimethanediyl)]tetrakis(phosphonic acid) (EDTMP)

EDTMP was synthesized from phosphorous acid, ethylenediamine and formaldehyde in the presence of HCl by a modified Mannich-type reaction [12]. To the stirring mixture of phosphorous acid (33.66 g) and conc. HCl (33.44 g) in a vessel under N_2_ atmosphere, ethylenediamine dihydrochloride (5 g) was added drop wise and heated to reflux. Then, aqueous solution of formaldehyde (37 %) is added drop wise to the mixture. Refluxing (at 100°C) is continued for 4h and the boiling suspension is then evaporated under vacuum. The residue was recrystallized from water/methanol mixture, m.p. 214–215˚C. IR (KBr, ν cm^−1^): 3308, 2633, 2311, 1668, 1436, 1356. ^1^H-NMR (D2O, δ ppm): 3.53 (d, J = 12.3 Hz, 8H,-N-CH_2_-P=O), 3.85 (s, 4H, -N-CH_2_-). ^13^C NMR (D_2_O, δ ppm): 51.63, 52.73. ^31^P NMR (D_2_O, δ ppm): 10.52.

### Radiolabeling of EDTMP with ^166^HoCl_3_

A stock solution of EDTMP was prepared by dissolution in 1 N NaOH and diluted to the appropriate volume with ultra pure water, to produce a solution of 50 mg/ml. For Labeling, an appropriate amount of the ^166^HoCl_3_ solution (0.1 ml, 50 mCi) containing the required amounts of activity was added to the desired amount of EDTMP solution (0.3 ml, 1–5 mg/ml). The complex solution was kept at room temperature for 45 min. The final solution was passed through a 0.22 μm membrane filter and pH was adjusted to 7–8.5 with 0.05 M phosphate buffer. Sterility and apyrogenicity of final preparation were ascertained by routine methods. The radiochemical purity was determined using Whatman 1 MM chromatography paper or ITLC-SG eluted with NH_4_OH: MeOH: H_2_O (0.2:2:4).

### Stability of ^166^Ho-EDTMP in final formulation

Stability of ^166^Ho-EDTMP in final preparation was determined by storing the final solution at 25ºC for 2 days and performing frequent ITLC analysis to determine radiochemical purity using Whatman 1 MM chromatography paper or ITLC-SG eluted with NH_4_OH: MeOH: H_2_O (0.2:2:4).

### Stability of ^166^Ho-EDTMP in presence of human serum

Final ^166^Ho-EDTMP solution (200μCi, 50 μl) was incubated in presence of freshly prepared human serum (300 μl) and kept at 37°C for 2 days. The stability was determined by performing frequent ITLC analysis using above mentioned chromatography system.

### Biodistribution studies

The biodistribution of Ho^3+^ cation as well as ^166^Ho-EDTMP were determined in wild-type rats. For each species, 100 μL (150 μCi) of radioactive solution was injected directly to normal rat through their caudal vein. The animals were sacrificed by ether asyxphycation at selected times after injection (2 to 48h) and percentage of injected dose in the tissues were determined with a γ-ray scintillation or a dose calibrator.

### Planar scintigraphy of ^166^Ho-EDTMP in wild-type rats

For imaging studies, ^166^Ho-EDTMP solution (7.4 MBq, 200 μl) was injected intravenously to male rats through their tail veins followed by propofol-xylazine mixture injection for anaesthetization. The images were acquired 4h after administration of the radio-pharmaceutical by a single-head SPECT system (Siemens) based on 80.6 keV peak (%15 energy window). The rat-to-septa distance was 12 cm.

## Conclusion

EDTMP ligand was synthesized in-house and the structure was determined using authentic spectroscopic methods followed by preparation and quality control of ^166^Ho-EDTMP (radiochemical purity>99%) using optimization studies. ^166^Ho-EDTMP and ^166^HoCl_3_ preparations were administered to wild-type rats and related biodistribution data were checked 2 to 24 h later showing at least 70% accumulation of the drug in the bone tissues. Scintigraphic images were taken from wild-type rats injected with ^166^Ho-EDTMP after 4 h and the biodistribution was shown to be consistent with scarification data. A comparative accumulation study for ^166^Ho-EDTMP and ^166^Ho^3+^ was performed for vital organs up to 48h. ^166^Ho-EDTMP is a promising agent for bone pain palliation therapy in skeletal metastases in human with low undesired dose to other organs in rodents.

## Figures and Tables

**Fig 1.**
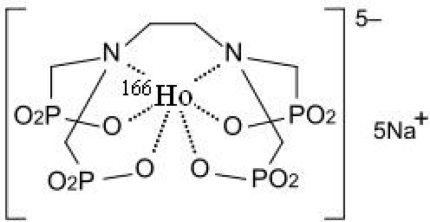
Chemical formula for ^166^Ho-EDTMP

**Fig. 6.**
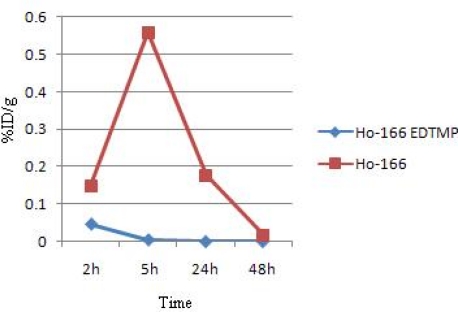
Comparative blood activity for ^166^Ho-EDTMP and ^166^HoCl_3_ in wild-type rats
